# Enhanced Thermal and Mechanical Properties of Cardanol Epoxy/Clay-Based Nanocomposite through Girard’s Reagent

**DOI:** 10.3390/polym16111528

**Published:** 2024-05-29

**Authors:** Ji Xu, Lingxiao Jia, Qixin Lan, Daheng Wu

**Affiliations:** 1 Key Laboratory of Advanced Marine Materials, Ningbo Institute of Materials Technology and Engineering, Chinese Academy of Sciences, Ningbo 315201, China; xuji2023@nimte.ac.cn (J.X.);; 2 Department of Ocean Engineering and Technology, College of Ocean, Zhejiang University, Hangzhou 310058, China

**Keywords:** bio-based epoxy resin, cardanol, Girard’s reagent, clay, nanocomposite

## Abstract

The green and environmentally friendly cardanol epoxy resin has a bright application prospect, but its insufficient thermal/mechanical properties seriously hinder its application. Adding nanoclay to polymer matrix is an effective method to enhance the thermal/mechanical properties of material, but the dispersion and compatibility of nanoclay in epoxy resin remain to be solved. In this work, active Girard’s reagent clay (PG-clay) and non-active Girard’s reagent clay (NG-clay) were prepared by using acethydrazide trimethylammonium chloride (Girard’s reagent) as the modifier, and cardanol epoxy resin/G-clay nanocomposites were synthesized by the “clay slurry composite method”. The results showed that both PG-clay and NG-clay were dispersed in the epoxy matrix in the form of random exfoliation/intercalation, which effectively improved the thermal/mechanical properties of the composites. Tg of the cardanol epoxy resin has raised from 19.8 °C to 38.1 °C (4 wt.% PG-clay). When the mass fraction of clay is 4%, the tensile strength of the non-reactive NG-clay increases by 128%, and the elongation at break also increases by 101%. Simultaneously, the active PG-clay can participate in the curing reaction of epoxy resin due to the amino group, forming a chemical bond between the clay layer and the resin matrix and establishing a strong interfacial force. The tensile strength of the composite is increased by 970%, and the elongation at break is also increased by 428%. This research demonstrates that the cardanol epoxy resin/G-clay nanocomposite stands as a highly promising candidate for bio-based epoxy resin materials.

## 1. Introduction

As one of the predominant thermosetting resins, epoxy resin (EP) is widely employed in various applications due to its excellent thermodynamic and mechanical properties, like the coating of polar icebreakers, where significant mechanical property is required [1,2,3,4,5]. Most of the global EP production currently relies on bisphenol A (BPA) as a raw material. However, the use of BPA in polymer synthesis has been explicitly prohibited in certain countries due to its well-established high toxicity and significant risks to human health [6,7,8,9,10,11]. To address these concerns, natural biomass phenols have been selected as potential substitutes for BPA. Cardanol, an extract derived from cashew nutshell oil, has garnered attention as a green bio-based alternative for the synthesis of EPs [12,13,14,15,16,17,18,19,20,21]. Typically, epoxy resin undergoes a chemical reaction with a curing agent to form a robust and highly crosslinked network, resulting in impressive thermal and mechanical properties [22,23]. Despite the promising applications of bio-based polymers, cardanol-based EPs exhibit certain limitations, such as inferior thermal and mechanical properties, which restrict their use in certain fields [12,13,14,15,16,24].

One established approach for enhancing the properties of polymers is the incorporation of nanoclay as a reinforcing agent [25,26,27,28,29,30,31,32,33]. To achieve the enhancement, it is essential that the clay is dispersed and exfoliated uniformly in the polymer matrix. When the clay is uniformly dispersed in the matrix, more interaction between interfaces can be formed. Meanwhile, a small amount of clay with exfoliation form can work as a hard segment in the matrix to improve the rigidity of the material. Accordingly, improving the dispersion of clay in polymers has been an early research objective in this field. The choice of organic modifier and the preparation process play crucial roles in achieving such uniform clay dispersion. Chen Bin [34] and colleagues successfully fabricated epoxy resin/clay nanocomposites with a randomly exfoliated structure using the “clay slurry composite method,” which effectively addressed the challenge of achieving uniform clay dispersion in the polymer matrix. However, it has been observed that achieving uniform clay dispersion alone provides limited enhancement to the composites. Building upon the foundation of uniform clay dispersion, enhancing the interfacial strength between the matrix and clay lamellae emerges as a critical factor for achieving composite performance enhancement [35,36,37,38,39,40]. In the EP/clay system, creating robust interfacial forces relies on the selection of organic modifiers that possess excellent compatibility with the EP matrix and contain reactive functional groups. Li Jingyu and colleagues [41] successfully produced epoxy/clay nanocomposites with a randomly exfoliated structure using dopamine hydrochloride as a clay modifier through the “clay slurry composite method”. The hydroxyl groups on dopamine clay were harnessed to establish hydrogen bonds with the EP matrix, thereby enhancing the interfacial strength between the clay and EP matrix. However, through the detailed characterization of thermodynamic and mechanical properties of EP/dopamine clay nanocomposites, it was found that the experimental results did not meet expectations, likely due to the relatively weak interfacial strength constructed by hydrogen bonds between dopamine clay and EP, resulting in limited enhancement of the composites.

To work out this limitation, some researchers explored the use of DMP-30 to modify the clay after partial protonation. The tertiary amine groups present on the organoclay were subsequently engaged in the curing reaction of EP, enabling the connection of clay lamellae with the EP matrix through chemical bonding. This approach offered the potential to construct a stronger interfacial force and significantly enhanced the composites. However, controlling the partial protonation of DMP-30 was challenging and difficult to implement. Consequently, the urgent task at hand in this field remains the development of methods to establish robust interfacial forces between the EP matrix and nanoclay, thus improving the overall strength of nanocomposites. Girard’s reagent is a remarkable compound characterized by the presence of a primary amine group and a quaternary ammonium ion [42,43]. The quaternary ammonium salt exhibits notable capability in conducting ion exchange reactions with the nanoclay, while the primary amine group displays reactivity toward the epoxy group. However, the susceptibility of the primary amine group to protonation limits its engagement in the curing reaction of the epoxy resin.

In this research, we explored the application of Girard’s reagent to modify the original clay and prepare a reactive organic clay capable of establishing a robust interfacial force between the clay lamellae and the material matrix. By employing the “clay slurry composite method”, we synthesized cardanol epoxy resin/G-clay (G-clay is the general name of PG-clay and NG-clay) nanocomposites. Comprehensive investigations were conducted to analyze the structural, mechanical, and thermomechanical properties of the G-clay/epoxy resin nanocomposites.

## 2. Materials and Methods

### 2.1. Materials

The cardanol-based epoxy resin (NC-514S) was acquired from Cardolite Corporation, RoadBristol, PA, USA. Girard’s reagent was procured from Shanghai Aladdin Biochemical Technology Co., Ltd., Shanghai, China. The epoxy curing agent (1618) was obtained from Guangzhou Jiuying Chemical Materials Co., Ltd., Guangzhou, China. Sodium nano montmorillonite with a specific surface area of 240 m^2^/g was sourced from Sud−Chemie, Munich, Germany. The cation exchange equivalent (CEC) of the montmorillonite was determined to be 92.6 meq/100 g.

### 2.2. Preparation of G-clay

For the preparation of NG-clay, 10 g of nanoclay was introduced into a 3000 mL glass beaker, and acetone was incrementally added along the beaker wall until the clay was fully wetted, forming a clay–acetone slurry. Subsequently, 2000 mL of deionized water was added to the beaker, and the mixture was vigorously stirred at 500 rpm for 2 h to obtain a clay suspension. To facilitate the ion exchange reaction, 12 g of Girard’s reagent was added, and the stirring was continued under a nitrogen atmosphere at 60 °C for 4 h, resulting in the formation of a white precipitate. The precipitate was separated by centrifugation at 7500 rpm for 5 min, yielding a clay slurry, which was subjected to repeated washing with alkaline deionized water followed by centrifugation. This washing–centrifugation cycle was repeated 5–6 times until no free Girard’s reagent was detected, and an NG-clay slurry was obtained. The solid content of the slurry was determined to be 11.8%, and it was subsequently stored in airtight containment at low temperatures.

To prepare PG-clay, a procedure similar to that of NG-clay was followed. Specifically, 10 g of nanoclay was mixed with acetone in a 3000 mL glass beaker to form a clay–acetone slurry. After adding 2000 mL of deionized water, the pH value of the solution was adjusted to remain above 12 throughout the system by slowly adding moderate NaOH solution and monitoring with a PH meter. The mixture was mechanically stirred at high speed for 2 h to achieve a clay suspension. The process employed for NG-clay was repeated to obtain a PG-clay slurry. The solid content of the PG-clay slurry was determined to be 12.93%.

### 2.3. Preparation of G-clay Nanocomposites

The G-clay was subjected to 3–4 washes with acetone to remove residual water, yielding a clay–acetone slurry. Subsequently, the clay–acetone slurry was mixed with NC-514S and introduced into a reaction flask with mechanical stirring. The mixture was then subjected to ultrasonic shaking for 1 h. The mixture was obtained by using a rotary evaporator until acetone was completely removed. A predetermined amount of epoxy resin curing agent 1618 was added, followed by mechanical stirring for 10 min. The bubbles were removed in a vacuum oven at 40 °C. The prepared mixture was then poured into PTFE molds and transferred to a blast-drying oven for curing. The curing conditions were as follows: 25 °C for 4 h, 60 °C for 12 h, 80 °C for 5 h, and finally, 120 °C for 5 h. A range of cardanol epoxy resin/G-clay nanocomposites with G-clay contents of 0 wt.%, 0.5 wt.%, 1 wt.%, 2 wt.%, and 4 wt.% were prepared.

### 2.4. Characterization

The chemical composition of G-clay was analyzed using X-ray photoelectron spectroscopy (XPS) on a multifunctional spectrometer (AXIS ULTRADLD, Kratos, Manchester, UK) equipped with Al Kɑ radiation. X-ray diffraction (XRD) patterns were obtained using a D8 ADVANCE instrument (Bruker, Karlsruhe, Germany) with Cu Kɑ radiation (*λ* = 0.154 nm). The experiments were conducted at an accelerating voltage of 40 kV and a current of 40 mA, scanning the range from 2° to 10° at a rate of 1° per minute. The changes in the interlayer spacing were calculated using the Bragg equation (2*d*sin*θ* = *nλ*). Fourier Transform Infrared Spectroscopy (FTIR) measurements were performed using a Micro-FTIR instrument (Agilent, Santa Clara, CA, USA) in the spectral range of 4000–400 cm^−1^, the sample preparation method is the KBr disc method, and the absorbance was calculated using the Lambert-Beer Law (*A* = a*bc*). Transmission Electron Microscopy (TEM) images were obtained using a JEM2100 instrument (JEOL, Tokyo, Japan). Tensile tests were conducted using an electronic universal testing machine (ZwickRoell, Ulm, Germany) with a load cell of 5 kN and a frequency of 500 Hz and samples were dumbbell-shaped with a size of 160 mm × 10 mm × 4 mm at a testing temperature of 25 °C and a tensile rate of 5 mm/min. Each group consisted of five samples, and the results were averaged. Dynamic mechanical analysis (DMA) was carried out on a DMA Q800 analyzer (TA, New Castle, DE, USA) under the conditions of −20 °C to 110 °C with a ramp rate of 3 k/min at 1 Hz. Differential scanning calorimetry (DSC) measurements were performed using a Perkin Elmer Diamond instrument (Perkin Elmer, Waltham, MA, USA) under a nitrogen atmosphere. The temperature program involved heating from −20 °C to 140 °C, holding for 3 min, cooling down to −50 °C, and then ramping up to 140 °C, all at a rate of 10 °C/min. The results were obtained by averaging multiple measurements. The glass transition temperature values of the test samples were determined based on the temperatures corresponding to the inflection points of the secondary heating curves.

## 3. Results

### 3.1. Preparation and Characterization of G-clay/Epoxy Nanocomposites

**Figure 1:**
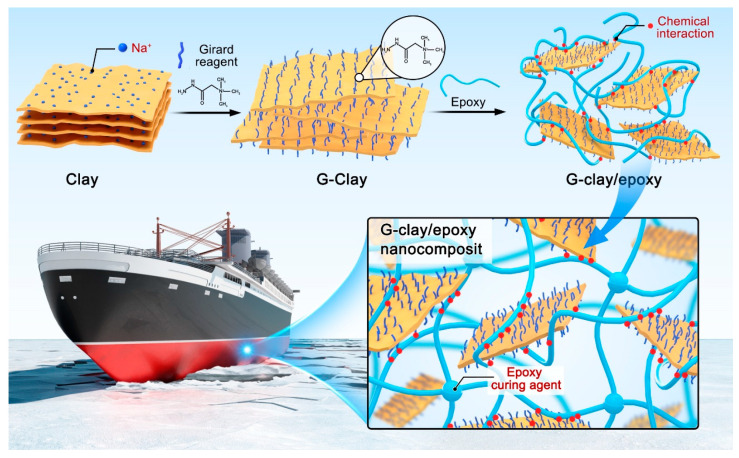
Preparation mechanism of G-clay/epoxy nanocomposites.

The preparation mechanism of G-clay nanocomposites can be demonstrated in Figure 1. The Girard’s reagent, bearing quaternary ammonium ions, facilitates an ion exchange process with sodium ions residing on the clay lamellae. This ion exchange results in the insertion of Girard’s reagent, leading to an expansion of the interlayer spacing between the lay lamellae, which will make it easier to be dispersed. When the G-clay is further mixed with cardanol epoxy resin, a comprehensive stripping process occurs, resulting in the disordered dispersion of the G-clay’s lamellar structure within the epoxy resin system. Simultaneously, the primary amine groups on Girard’s reagent establish chemical bonds with cardanol epoxy resin, fostering a robust interfacial interaction between the clay lamellae and the resin matrix. In the final step, the addition of a curing agent initiates the crosslinking of cardanol epoxy resin. The resulting G-clay/epoxy nanocomposites exhibit promise for application in the field of bio-based epoxy resins, particularly in scenarios demanding enhanced mechanical and thermal properties.

**Figure 2:**
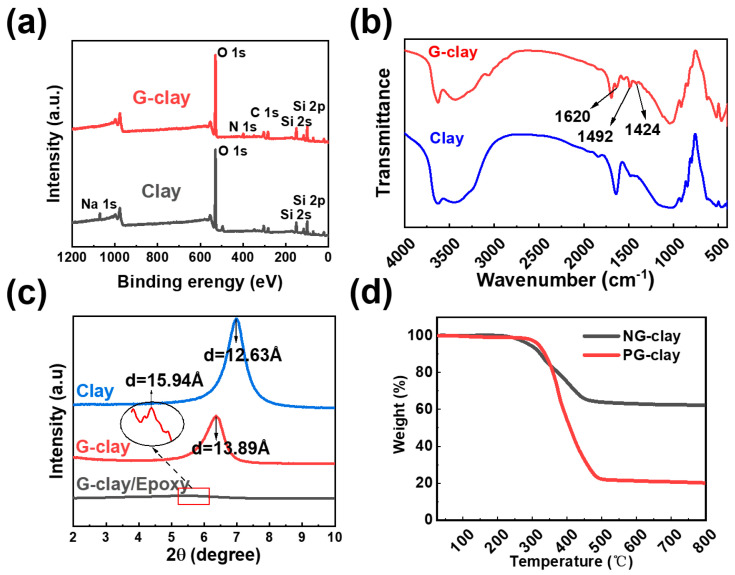
(**a**) XPS full-spectrum scan of original clay and G-clay; (**b**) FTIR spectra of the original clay and G-clay; (**c**) XRD patterns of clay, G-clay, G-clay/epoxy; (**d**) TGA curves of PG-clay–EP and NG-clay–EP.

X-ray photoelectron spectroscopy (XPS) was employed to characterize both the original clay and G-clay samples. Figure 2a illustrates the XPS electron spectra obtained from the original clays, revealing distinctive peaks corresponding to silicon (Si 2s), oxygen (O 1s), and sodium (Na 1s) atoms following a comprehensive spectral scan [41]. In Figure 2a, the XPS electron spectra of G-clay are presented. Upon introducing Girard’s reagent into the clay lamellae, characteristic peaks attributed to carbon (C 1s) and nitrogen (N 1s) atoms, which are intrinsic to the Girard’s reagent, emerged in the full-spectrum scan of G-clay [44]. Notably, the characteristic peaks originating from the sodium (Na 1s) atom derivatives were absent. This observation implies the complete replacement of Na+ ions in the interlayer spaces of the original clay by Girard’s reagent. Thus, these findings strongly suggest a comprehensive and reacted ion exchange between the clay and Girard’s reagent.

Figure 2b presents the FTIR spectra of the original clay and G-clay. The original clay exhibits prominent characteristic peaks in the curve at 1034 cm^−1^, corresponding to the Si-O bonds within the clay structure [36,45]. Additionally, a characteristic peak related to the stretching frequency of -OH is observed at 3636 cm^−1^ and 1640 cm^−1^ in the curve [36,45]. This peak can be attributed to the adsorption of moisture from the ambient air onto the clay surface. In comparison, the FTIR spectrum of G-clay displays a newly observed characteristic peak at 1620 cm^−1^, distinguishing it from the original clay. This peak is assigned to the stretching vibration of primary amines present in the Girard’s reagent incorporated within the G-clay [44]. Characteristic bands at 1492 cm^−1^ and 1424 cm^−1^ can be attributed to the asymmetric and symmetric bending of the methyl group of Girard’s reagent [43]. These observations provide further confirmation that Girard’s reagent undergoes an ion exchange reaction with Na+ ions present between the clay lamellae, resulting in its successful insertion into the clay structure.

Figure 2c displays the XRD patterns of the original clay, G-clay–acetone slurry, and cardanol epoxy/G-clay nanocomposites. The changes of lamellar spacing are calculated by 2θ according to the Bragg equation [45]. The original clay exhibits a prominent characteristic peak at 2*θ* = 6.9°, corresponding to a lamellar spacing of 12.63 Å. Following the modification with Girard’s reagent, the G-clay–acetone slurry reveals a new characteristic peak at 2*θ* = 6.35°, indicating a clay lamellar spacing of 13.89 Å. This signifies the successful insertion of Girard’s reagent into the clay lamellae through the ion exchange reaction.

Analysis of the XRD patterns for the cardanol epoxy/G-clay nanocomposites demonstrates a continuous shift of the diffraction peaks associated with the (001) plane toward smaller angles. Additionally, a low-intensity peak appears at 2*θ* = 5.56°, indicating complete disruption of the ordering of G-clay lamellae within the cardanol epoxy resin [36]. Furthermore, this expansion of the clay lamellae spacing suggests the successful integration of G-clay within the resin matrix, where the G-clay has been effectively dispersed.

Unprotonated Girard’s reagent-modified clay (PG-clay) can engage in the curing reaction and establish a robust chemical interfacial interaction with the epoxy matrix. To demonstrate that, we conducted experiments involving PG-clay and NG-clay with excess epoxy resin NC-514S under identical curing conditions. The resulting reaction products were subsequently subjected to extraction in a Soxhlet apparatus using xylene for 48 h to remove any unreacted epoxy resin. Following this, the remaining products were carefully dried in an oven at 60 °C. The resulting dried product was then subjected to thermal gravimetric analysis (TGA) to generate the heat loss profile presented in Figure 2d.

The TGA curve for NG-clay manifests a heat loss of only 37.69% at 800 °C, predominantly attributed to the thermal decomposition of the organic modifier: Girard’s reagent. In contrast, the TGA curve for PG-clay exhibits a more significant heat loss of 79.85% at 800 °C, indicating a higher content of organic matter within the reaction product. Thus, it can be inferred that PG-clay possesses the reactive property necessary for interaction with the epoxy resin matrix. Hence, our findings demonstrate that unprotonated Girard’s reagent-modified clay (PG-clay) exhibits reactivity and is capable of participating in the curing reaction. Consequently, a chemical bond is formed between the clay lamellae and the epoxy resin matrix, facilitating the establishment of a chemical interfacial interaction.

**Figure 3:**
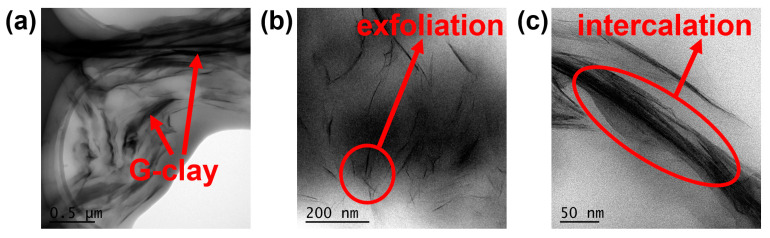
TEM images of G-clay/epoxy nanocomposites with 4 wt% G-clay at magnification of (**a**) 6000×, (**b**) 20,000× and (**c**) 50,000×.

Transmission electron microscopy (TEM) unveiled the distribution of G-clay within the bio-based epoxy resin matrix, as depicted in Figure 3. Uniform exfoliation and dispersion of clay are crucial to enhance the thermodynamic and mechanical properties, while a certain intercalation is also necessary [36]. The dark regions correspond to the G-clay lamellae, as shown in Figure 3a, upon low-magnification examination. Compared with other composites that use clay as intercalation, G-clay/epoxy nanocomposites exhibited a remarkable and uniform dispersion throughout the bio-based epoxy resin matrix without apparent agglomeration or cavitation [45]. Figure 3b,c provides a high-magnification view of the clay layer dispersion, clearly demonstrating that a majority of the G-clay was dispersed in the form of random exfoliation within the bio-based epoxy resin matrix. Only a small fraction displayed intercalation, resulting in an overall structure showcasing a mixed configuration of random exfoliation/intercalation. The excellent dispersion of G-clay can be attributed to the inherent suitability of the Girard reagent for the preparation of the “Clay Slurry.” Moreover, the dispersion of G-clay owes its efficacy to the driving force exerted by the reactive amine groups involved in the curing reaction.

### 3.2. Effect of Organic Modifiers on Thermo-Mechanical Properties of Nanocomposites

**Figure 4:**
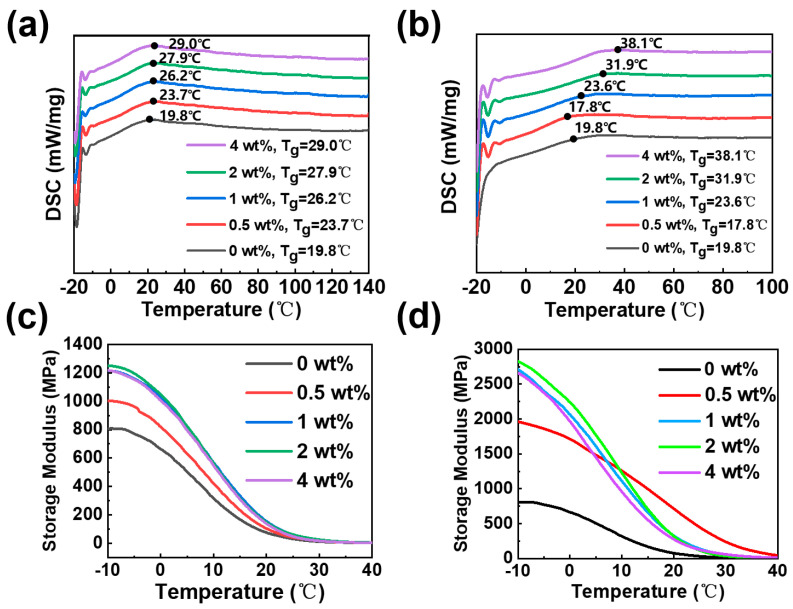
DSC curves of (**a**) NG-clay/cardanol-based epoxy composites and (**b**) PG-clay/cardanol-based epoxy composites; Dynamic mechanical properties of (**c**) NG-clay/cardanol-based epoxy composites and (**d**) PG-clay/cardanol-based epoxy composites.

Figure 4a depicts the differential scanning calorimetry (DSC) curves obtained for pure cardanol epoxy and cardanol epoxy/NG-clay nanocomposites containing varying NG-clay contents. This analysis aimed to explore the influence of NG-clay components on the glass transition temperature (Tg) of the cardanol epoxy/NG-clay nanocomposites. The DSC curves reveal that the Tg of the pure cardanol epoxy resin was 19.8 °C. However, upon the addition of 0.5 wt.% NG-clay, the Tg of the composites increased to 23.7 °C, with further enhancements observed as the NG-clay content increased. Remarkably, the Tg of the composite material soared to 29 °C when 4 wt.% NG-clay was incorporated, representing a notable 9.2 °C rise compared to the pure bio-based epoxy resin. Figure 4b presents the DSC plots of cardanol resin/PG-clay nanocomposites with varying contents. The curves demonstrate that the Tg temperatures of PG-clay composites at 0.5 wt.% and 1 wt.% are lower than those of NG-clay composites. However, as the PG-clay content increases, the glass transition temperatures surpass those observed in NG-clay composites at both 2 wt.% and 4 wt.% contents. Notably, at 4 wt.% PG-clay content, the glass transition temperature elevated to 38.1 °C, a substantial 18.3 °C above that of the pure bio-based epoxy resin.

The glass transition temperature (Tg) of the cardanol epoxy resin/G-clay nanocomposites was significantly influenced by varying clay contents. This phenomenon can be attributed to the flexible nature of NC-514S, an epoxy resin with a low crosslinking density in its cured state. Introducing PG-clay with reactive properties led to a gradual increase in the crosslinking degree of the composites. The main factors affecting the glass transition temperature of nanocomposites are not only the toughness and crosslinking density of the polymer itself but also the dispersion degree and the interfacial strength of the nano-filler. The PG-clay was dispersed within the matrix in a random exfoliated manner. Furthermore, the primary amine present in the Girard’s reagent actively engaged in the curing reaction of the epoxy resin. This interaction improved the interfacial strength of the composite and constrained the mobility of the molecular chains within the bio-based epoxy resin. Consequently, the Tg of the material exhibited a noteworthy increase.

The impact of G-clay on the thermomechanical properties of the nanocomposites was assessed through dynamic mechanical analysis (DMA). As illustrated in Figure 4c, the energy storage modulus of the original cardanol epoxy resin was measured to be 800 MPa. However, when 2 wt.% of NG-clay was introduced, the energy storage modulus peaked at 1250 MPa. This significant enhancement can be attributed to the homogeneous dispersion of clay lamellae within the nanometer-scale cardanol epoxy resin matrix.

Figure 4d demonstrates that the maximum energy storage modulus of 2811 MPa was achieved when 2 wt.% of PG-clay was incorporated. This remarkable increase can be attributed not only to the random dispersion of PG-clay within the epoxy resin matrix but also to the presence of reactive amino groups on the PG-clay. These amino groups actively participate in the curing reaction of the epoxy resin, promoting the formation of strong interfacial forces between the reinforcing body (clay) and the resin matrix. Consequently, the energy storage modulus of the material is significantly enhanced.

It is noteworthy that as the NG-clay content increases to 4 wt.%, the energy storage modulus of the composites starts to decline, reaching 1216 MPa. Similarly, with a PG-clay content of 4 wt.%, the energy storage modulus decreases to 2629 MPa. This observation confirms the consistency of these properties across the different clay.

**Figure 5:**
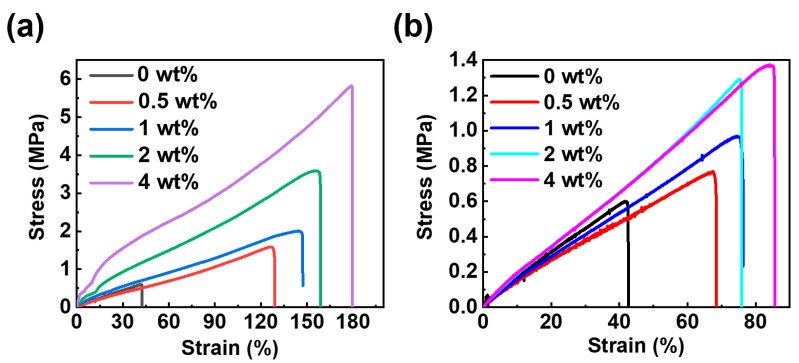
Stress–strain curves of (**a**) bio-based epoxy/NG-clay nanocomposites and (**b**) bio-based epoxy/PG-clay nanocomposites.

Figure 5a presents the stress–strain curves of the pure cardanol epoxy resin and cardanol epoxy resin/NG-clay nanocomposites with varying clay contents. The corresponding tensile data are summarized in Table 1. The pure cardanol resin exhibits a tensile strength of 0.60 MPa and a Young’s modulus of 13.76 MPa. With the increase in NG-clay content, the tensile strength and modulus of the composite are improved to some extent.

Upon incorporating 0.5 wt.% NG-clay, the tensile strength of the composites increases to 0.77 MPa, accompanied by a corresponding modulus of 28.34 MPa. At the highest NG-clay content of 4 wt.%, the composites achieve a maximum tensile strength of 1.37 MPa, representing a 128% enhancement compared to the pure bio-based epoxy resin. Concurrently, the modulus of the composite material reaches 60.50 MPa.

In contrast, the inclusion of reactive PG-clay contributes significantly to the mechanical properties of the epoxy resin in Figure 5b, and the corresponding tensile data are summarized in Table 2. At 4 wt.%, the tensile strength reaches 5.82 MPa, exhibiting a striking 970% improvement over the pure bio-based epoxy resin. Meanwhile, the modulus of the composite reaches 546.90 MPa, which is an extraordinary increase of 3974% compared to the pure bio-based epoxy resin.

The incorporation of G-clay into the cardanol epoxy resin resulted in substantial improvements in both tensile strength and modulus, surpassing the values obtained before modification. This improvement can be attributed to the dispersion of G-clay within the cardanol epoxy resin, occurring in the form of random exfoliation. This dispersal mechanism significantly increased the interfacial area between the phases.

However, through the comparison between Table 1 and Table 2, it can be seen that the mechanical properties of PG-clay/epoxy nanocomposites are much better than NG-clay. When the mass fraction is 4%, the tensile strength of PG-clay/epoxy nanocomposites is 424.82% of NG-clay/epoxy nanocomposites. Such a large difference is because PG-clay retained active amino groups that actively participated in the curing reaction of EP. As a result, a strong interfacial force was established between the nanoclay and the cardanol epoxy resin matrix, thereby enhancing the interfacial strength of the nanocomposites. Consequently, a significant enhancement in tensile strength and modulus was observed within the materials.

In particular, the inclusion of G-clay not only enhanced the tensile strength and modulus of the bio-based epoxy resin but also resulted in a substantial increase in the elongation at the break of the composites. For the pure cardanol epoxy resin, the elongation at break measured 41.79%. However, when NG-clay was added at a mass fraction of 4%, the elongation at break of the bio-based nanocomposites reached 84.26%, representing a 101% increase compared to the pre-modification levels. When PG-clay was added at the same mass fraction of 4%, the elongation at break of the bio-based nanocomposites rose to 178.82%, exhibiting an impressive 428% increase.

This considerable enhancement in elongation at break can be attributed to the inherent characteristics of the cardanol epoxy resin, NC-514S, which is known for its flexibility and relatively weak cohesion in the cured state, evidenced by its modest tensile strength of 0.60 MPa. Under external elongation, the material undergoes minimal deformation before experiencing failure, resulting in low elongation at break values. When G-clay was dispersed in cardanol epoxy resin, the deformability of the interfaces increased, resulting in an increase in elongation at break.

Nevertheless, the elongation at the break of PG-clay/epoxy nanocomposites is 212.22% of NG-clay/epoxy nanocomposites. Unlike NG-clay, the introduction of reactive PG-clay engenders increased physical crosslinking points within the chain of the flexible cardanol epoxy resin. This augmentation in crosslinking density significantly strengthens the cohesion of the composite material, allowing for greater deformation before failure occurs. Therefore, as the tensile strength of the cardanol epoxy resin/PG-clay nanocomposites increases, so does their elongation at break.

## 4. Conclusions

In this paper, two types of cardanol epoxy resin/G-clay nanocomposites were prepared by using Girard’s reagent as an organic modifier to establish robust chemical interfacial interactions. This novel bio-based epoxy composite shows excellent thermodynamic and mechanical properties due to the random exfoliation and uniform dispersion of G-clay. PG-clay effectively preserved the primary amine groups, enabling their participation in the curing reaction of the epoxy resin. This facilitated the establishment of robust chemical interfacial interactions, leading to a huge increase in thermal and mechanical properties. Compared to the glass transition temperature (Tg) of the pure bio-based epoxy resin, which was measured at 19.8 °C, the Tg value of cardanol-based epoxy resin/PG-clay nanocomposites increased to 38.1 °C at 4 wt.% PG-clay content. The mechanical properties of the cardanol-based epoxy resin/PG-clay nanocomposites exhibited the most significant improvement. With a PG-clay content of 4 wt.%, the composites displayed a remarkable increase in tensile strength by 970%, modulus by 3974%, and elongation at the break by 428%. This research presents promising opportunities for the development of advanced bio-based epoxy resin nanocomposites. Future studies can focus on more detailed mechanisms of interfacial interactions. 

## Figures and Tables

**Table 1:** Mechanical properties of NG-clay/cardanol-based epoxy composites.

Content of NG-clay (wt.%)	Tensile Strength (MPa)	Young’s Modulus (MPa)	Elongation at Break (%)
0	0.60 ± 0.06	13.76 ± 3.26	41.79 ± 1.32
0.5	0.77 ± 0.17	28.34 ± 5.58	67.50 ± 2.28
1	0.96 ± 0.24	38.93 ± 6.65	74.75 ± 2.36
2	1.29 ± 0.22	48.11 ± 8.24	75.19 ± 1.68
4	1.37 ± 0.28	60.50 ± 8.53	84.26 ± 3.56

**Table 2:** Mechanical properties of PG-clay/cardanol-based epoxy composites.

Content of PG-clay (wt.%)	Tensile Strength (MPa)	Young’s Modulus (MPa)	Elongation at Break (%)
0	0.60 ± 0.06	13.76 ± 3.26	41.79 ± 1.32
0.5	1.58 ± 0.05	100.50 ± 5.46	126.91 ± 11.82
1	2.00 ± 0.11	161.91 ± 7.91	144.35 ± 22.07
2	3.59 ± 0.23	304.90 ± 8.86	155.25 ± 17.33
4	5.82 ± 0.54	546.90 ± 12.53	178.82 ± 3.05

## Data Availability

Data are contained within the article.
